# Passive Sampler for Measurements of Atmospheric Nitric Acid Vapor (HNO_3_) Concentrations

**DOI:** 10.1100/tsw.2001.323

**Published:** 2001-12-05

**Authors:** Andrzej Bytnerowicz, Pamela E. Padgett, Michael J. Arbaugh, David R. Parker, David P. Jones

**Affiliations:** ^1^ USDA Forest Service, Pacific Southwest Research Station, 4955 Canyon Crest Drive, Riverside, California 92507, USA; ^2^ University of California, Department of Environmental Sciences, Riverside, California 92521, USA

**Keywords:** air pollution, nitric acid vapor, passive sampler, monitoring, remote sites

## Abstract

Nitric acid (HNO_3_) vapor is an important nitrogenous air pollutant responsible for increasing saturation of forests with nitrogen and direct injury to plants. The USDA Forest Service and University of California researchers have developed a simple and inexpensive passive sampler for monitoring air concentrations of HNO_3_. Nitric acid is selectively absorbed on 47-mm Nylasorb nylon filters with no interference from particulate NO_3_^-^. Concentrations determined with the passive samplers closely corresponded with those measured with the co-located honeycomb annular denuder systems. The PVC protective caps of standardized dimensions protect nylon filters from rain and wind and allow for reliable measurements of ambient HNO_3_ concentrations. The described samplers have been successfully used in Sequoia National Park, the San Bernardino Mountains, and on Mammoth Mountain in California.

## INTRODUCTION

Nitric acid ($HNO_3$) vapor, an air pollutant found in photochemical smog, affects the health of people and vegetation. Because of its very high reactivity and deposition velocity[1], $HNO_3$ provides large amounts of nitrogen (N) to forests and other ecosystems[2]. For example, in forests of the Los Angeles Basin mountain ranges, $HNO_3$ provides more that 60% of the dry-deposited N to mixed conifer forests[3]. Increased deposition of $HNO_3$ may lead to eutrophication of sensitive ecosystems and contamination of surface water with nitrate ($NO_3^-$), especially in locations close to photochemical smog source areas such as mountain ranges in southern California close to Los Angeles[4]. While ambient concentrations of $HNO_3$ expressed as 24-h averages did not exceed 2.1 $\mu g/m^3$ in a rural European location[5] and 4.8 $\mu g/m^3$ as a 12-h daytime average in the Sierra

Nevada mountains in California[6], in the San Gabriel Mountains of southern California the 12-h daytime concentrations reached 28 μg/m$^3$[7]. Information on $HNO_3$ spatial and temporal distribution in forest ecosystems of the western U.S. is essential for a better under-standing of its phytotoxic potential and development of N deposition models at a landscape level.

Measurements of $HNO_3$ concentrations using active sampling, such as annular denuder systems[8] or honeycomb denuder systems[9], although precise, are expensive and labor intensive. Use of these systems on a larger scale (landscape) is very difficult even if battery-operated systems are utilized. Similarly to other pollutants, such as ozone, there is a clear need for simple and inexpensive device which would allow for landscape-level monitoring of the pollutant, including remote locations.

Although $HNO_3$ may be toxic to plants at elevated concentrations[10], its role as a source of N in different ecosystems is of primary importance. While for evaluation of toxic effects, peak concentration values of pollutants have to be known, for estimates of deposition, information on average concentrations of $HNO_3$ and its deposition velocity may be sufficient. Results from passive samplers, which integrate concentrations over a collection period, provide such information. Passive samplers, devices that are relatively inexpensive, low-maintenance, and do not require electric power, can be easily used for large-scale monitoring networks providing valuable information for the development of landscape-level pollutant distribution models.

## EXPERIMENTAL METHODS

Nylon filters of 47 mm diameter (Nylasorb, Pall Corporation) have been used as a collection medium for $HNO_3$. Such filters have been routinely used in annular denuder systems and filter packs for collection of this pollutant[11]. The filters were mounted in PVC double rings of 39 mm inner diameter (total surface of the exposed portion of a filter on both sides = 0.002389 m$^2$). Evaluation of filter responses to changing levels of $HNO_3$, absorption capacity of filters, and performance of various protective caps was done in the continuously stirred tank reactors (CSTR) with controlled concentrations of the pollutant[12]. For testing the absorbing capacity of nylon filters, 30 of those mounted in the PVC rings were exposed to controlled high concentrations of $HNO_3$ in the CSTR chamber. Every 24 h, three replicate filters were removed from the chamber during 240 h of exposures. For calibration, additional exposures of nylon filters (PVC double rings without caps) were performed at low doses of the pollutant. Calibration curve was developed based on the combined CSTR results below the filter saturation point. Concentrations of $HNO_3$ and particulate $NO_3$ in CSTR chambers were monitored with honeycomb denuder/filter pack systems operating at 10 L/min flow rate, which provided 2.2 μm cutoff point for particulate matter[9]. Honeycomb denuder/filter pack systems were changed at the same time as passive samplers.

Preliminary tests indicated that, due to high reactivity of $HNO_3$ with surfaces, no pre-screens or diffusion tubes for controlling rate of air moving to sampling media could be used (no detectable amounts of the pollutant reached the nylon filters). Instead, $HNO_3$ was collected on filters well protected from rain and wind by PVC caps, and calibrated against the co-located honeycomb denuder systems. Tests of several designs of protective caps for nylon filters mounted in the PVC rings were performed in the CSTR chambers.

After exposures, nylon filters were placed in 250 ml Erlenmeyer flasks into which 20 mL of distilled, deionized water was added. The flasks were shaken on a wrist-action shaker for 15 min at middle speed. Amount of absorbed $HNO_3$ was determined as $NO_3^-$ with ion exchange chromatograph (Dionex Model 4000i). Results of the absorbed $NO_3^-$ were expressed as mg $NO_3^-/m^2$ of the total filter surface (two sides). Denuders and filters from honeycomb denuder/filter pack systems were extracted in 0.0024 *M* $Na_2CO_3$ + 0.0030 *M* $NaHCO_3$ solution. Concentrations of $NO_3^-$ were also determined with ion exchange chromatography (Dionex Model 4000i).

Field tests of samplers were done in Sequoia National Park of the western Sierra Nevada, California. In summer 1999, passive samplers for $HNO_3$ and honeycomb denuder/filter pack systems were located at five monitoring sites: Ash Mountain, 504 m; Crystal Cave, 1,617 m; Lower Kaweah, 1,905 m; Marble Fork, 2,025 m; and Wolverton, 2,207 m along the Kaweah/Marble Fork drainage in Sequoia National Park. The Kaweah/Marble Fork drainages are directly exposed to airflow from the San Joaquin Valley in California.

Student's *t* test was used for comparison of performance of passive samplers against active monitors/honeycomb denuder systems. Differences are reported as significant at $p < 0.05$[13].

## RESULTS AND DISCUSSION

Deposition of $HNO_3$ to nylon filters (measured as $NO_3^-$) was proportional to the $HNO_3$ dose in the CSTR exposure chambers up to about 170 mg $NO_3^-/m^2$ (double-sided area of nylon filter). Above that level, nylon filters were not absorbing $NO_3^-$ linearly indicating near saturation point with $HNO_3$ ($HNO_3$ dose of about 5700 $\mu g/m^3 \times h$). These results indicate that the open-ring filters would saturate after 14 days of exposure to average concentration of $HNO_3$ of about 17 $\mu g/m^3$ (6.6 ppb). A strong linear relationship between concentrations of the pollutant and its deposition to nylon filters allowed for precise calibration of passive samplers (Fig. 1). While absorption of $NO_3^-$ by nylon filters was closely correlated with $HNO_3$ ambient concentrations ($R^2 = 0.9954$), no relationship was found with the ambient concentrations of particulate $NO_3^-$ (Fig. 2).

The following formulas for calculating $HNO_3$ concentrations with passive samplers have been developed:

$$NO_3^- \text{ deposition to nylon filters (mg/m}^2) = (c_{NO3^-} \text{ of a filter extract} - c_{NO3^-} \text{ of a blank; mg } NO_3^-/L) \times 0.02 \text{ L}/0.002389 \text{ m}^2 \quad (1)$$

$$HNO_3 \text{ dose } (\mu g/m^3 \times h) = K \times NO_3^- \text{ deposition (mg/m}^2) \quad (2)$$

$$HNO_3 \text{ concentrations } (\mu g/m^3) = HNO_3 \text{ dose } (\mu g/m^3 \times h) / \text{exposure time (h)} \quad (3)$$

where K values (slope of calibration curves) for various protective caps are listed in Table 1.

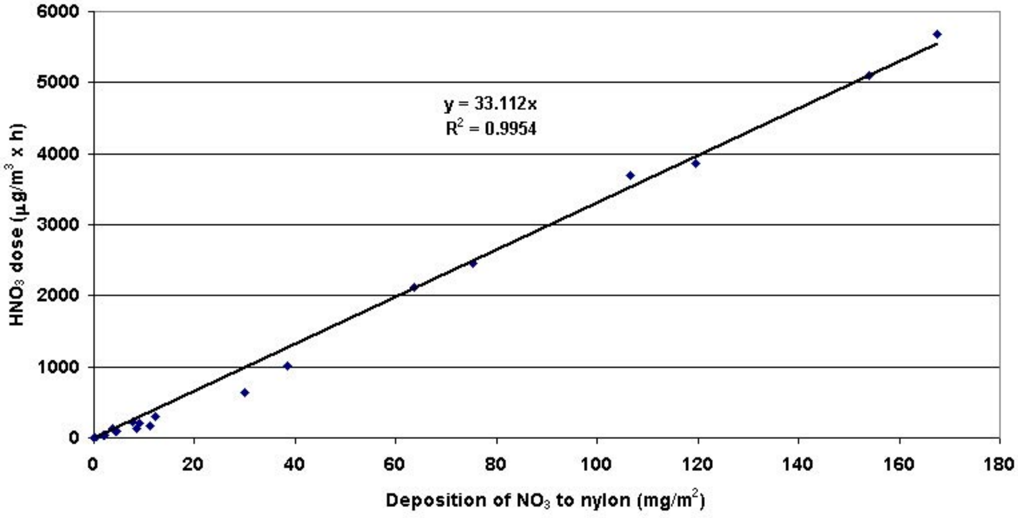

**FIGURE 1.** Calibration of nylon filters as collecting medium in the $HNO_3$ passive samplers. Calibration curve was developed in the CSTR chambers with controlled levels of $HNO_3$.

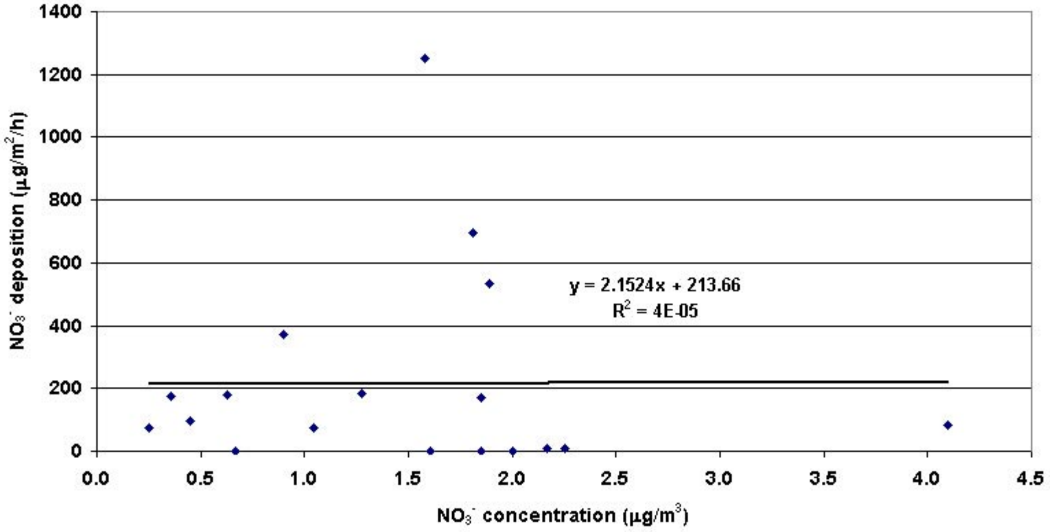

**FIGURE 2.** Ambient concentrations of particulate $NO_3^-$ and rates of $NO_3^-$ deposition to nylon filters were not correlated.

**TABLE 1**
**Characteristics of Tested Protective Caps and K Values (Slope of Calibration Curves) for $HNO_3$ Concentration Calculations**

| Protective Cap Type | Inner Diameter of a Cap (mm) | Distance between Center of Filter and Bottom Rim of Cap (mm) | Number of Filters in Cap | K Value |
|---|---|---|---|---|
| Open ring without a cap | | | | 33.11 |
| Forest Service cap | 90 | 42 | 1 | 49.94 |
| UC Riverside short cap | 107 | 37 | 2 | 62.47 |
| UC Riverside long cap | 101 | 247 | 2 | 315.33 |

Protective caps reduced the rate of $HNO_3$ uptake by nylon filters and increased the K values (Table 1). These tests indicated that enclosure of filters in caps of various designs allows for $HNO_3$ collection at higher concentrations or longer collection periods.

Field tests in Sequoia National Park indicated that the highest $HNO_3$ concentrations determined both with passive samplers and honeycomb denuders were recorded at 504 m altitude at Ash Mountain, and the lowest concentrations were at 2,207 m altitude at Wolverton. Ranges of $HNO_3$ concentrations determined with honeycomb denuder systems and passive samplers were similar (0.6 – 2.1 and 0.4 – 2.9 µg/m$^3$, respectively) (Fig. 3). Statistical evaluation of the $HNO_3$ data indicated that in four out of five sites where both honeycomb denuder systems and passive samplers were used, concentrations did not significantly differ. This suggests that these $HNO_3$ passive samplers could be used for estimates of ambient levels of the pollutant in remote mountain locations. However, due to different collection regimes (2-week-long integrated values for passive samplers versus two 24-h measurements per month during the growing season with honeycomb denuder systems), such a comparison has to be viewed with caution. These results also indicate that two different sampling regimes and monitoring methods provided very similar estimates of $HNO_3$ contamination in the study area. Precision of measurements with the $HNO_3$

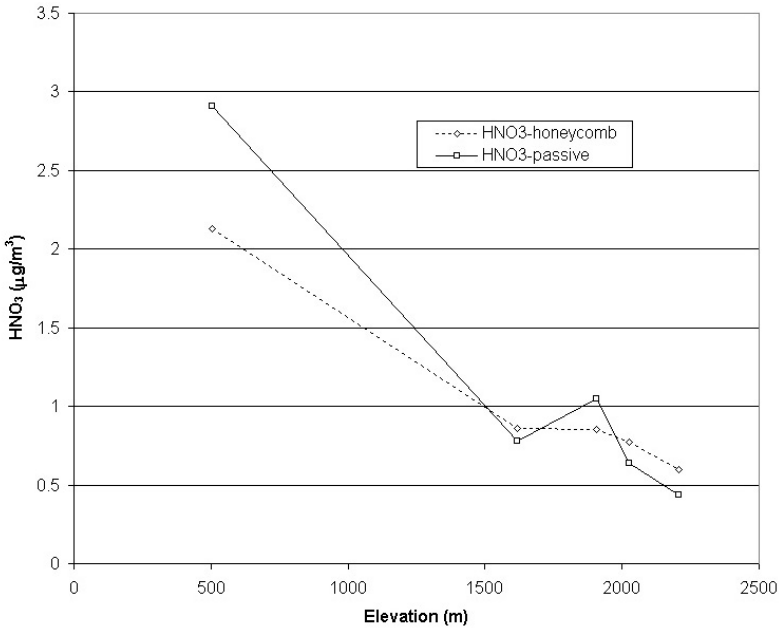

**FIGURE 3.** Changes of seasonal average $HNO_3$ concentrations measured with honeycomb denuder/filter pack systems and passive samplers along the Kaweah/Marble Fork drainage in Sequoia National Park in summer 1999.

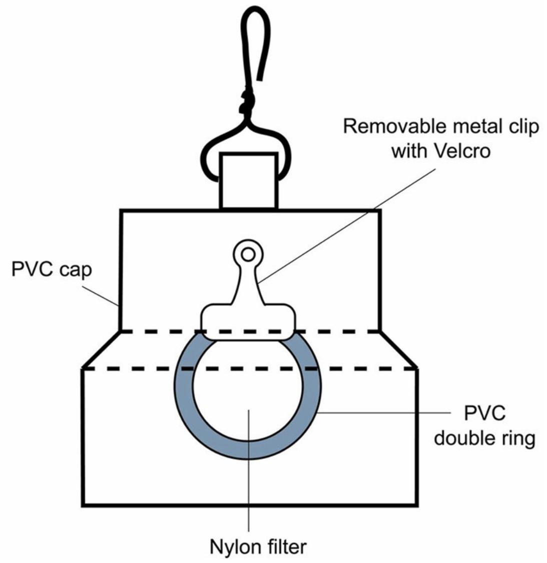

**FIGURE 4.** Schematic of $HNO_3$ protective cap designed at the University of California, Riverside. Two nylon filters enclosed in PVC double rings were inserted in metal clips that were attached to the cap's inner wall with Velcro tape.

passive samplers was good — the coefficient of variation of three replicate measurements stayed between 0.8 and 25.4% (average 9.2%) during the entire monitoring period. Precision of $HNO_3$ passive samplers could be additionally improved if a diffusion barrier providing uniform flow of air to the collection medium could be used.

A protective cap developed at the Department of Environmental Sciences of the University of California (UC-Riverside short cap) provided good protection of nylon filters against wind and rain (Fig. 4). This type of a protective cap has been successfully used in monitoring studies in remote mountain ranges of the San Bernardino and Mammoth Mountains. Results of these experiments are presented in other papers published in this volume [14,15].

In summary, it can be said that the developed sampler provides much-needed information regarding ambient levels of $HNO_3$ vapor on a large scale. However, in order to improve performance and reliability of the sampler, especially in conditions of high winds (a situation quite typical for these mountain environments), additional tests of diffusion barriers controlling flow of air to the collecting medium are needed. The USDA Forest Service scientists will perform such tests during the 2001 summer season.

## CONCLUSIONS

1. A strong relationship between ambient concentrations of $HNO_3$ and nitrate collected to nylon filters was determined in the CSTR chambers. The nylon filters used in this study (effective double-sided area of 0.002389 $m^2$) have a capacity of collecting 5,700 $\mu g$ $HNO_3/m^3 \times h$ which allows for long-term (several weeks) monitoring of the pollutant. Protective caps of various designs reduce rate of $HNO_3$ deposition and amount of nitrate absorbed on the collecting medium and extend potential time of $HNO_3$ collection.
2. Field tests demonstrated that results obtained with passive samplers were similar to the values recorded with honeycomb annular denuder/filter pack systems. Therefore, we conclude that passive samplers can reliably measure ambient levels of $HNO_3$.
3. Low price, simple design, and easy operation of the samplers allow for a large (landscape) level monitoring of $HNO_3$. This offers a possibility for evaluation of risks to ecosystems and humans.
4. Additional tests are needed for improving performance of the samplers. Enclosing nylon filters in collection devices providing steady airflow by diffusion barriers will be tested.

## ACKNOWLEDGEMENTS

Diane Alexander and Enrique Jimenez of the USDA Forest Service Pacific Southwest Research Station helped with chemical analyses. USDA Grant No. 97-35100-4402 supported tests of passive samplers in CSTR chambers. The National Park Service and the U.S. Environmental Protection Agency supported tests of passive samplers in Sequoia National Park.

## BIOSKETCHES

**Andrzej Bytnerowicz** is an Ecologist with the USDA Forest Service, Pacific Southwest Research Station in Riverside, California. He received a Ph.D. in Natural Sciences in 1981 from Silesian University in Katowice, Poland and an M.S. in Food Chemistry in 1972 from Warsaw Agricultural University, Poland. His research interests include development and tests of air pollution monitoring techniques for remote locations, development of models for air pollution distribution and deposition in mountain forest ecosystems, coordination of studies on effects of ozone and nitrogenous air pollutants in forests of California and Central Europe, investigations of effects of air pollutants on plant antioxidant systems.

**Pamela E. Padgett** is a Research Plant Physiologist with the USDA Forest Service, Pacific Southwest Research Station in Riverside, California. She received a Ph.D. in Botany in December 1993 from the University of California, Riverside and a B.S. in Agronomy in September 1987 from Virginia Polytechnic Institute and State University, Blacksburg, Virginia. Her research interests are focused on understanding the fundamental mechanisms driving plant responses to air pollution and other anthropogenic disturbances. Current studies range from, laboratory studies of nitric acid (a common urban pollutant) uptake and assimilation by plant foliage, atmospheric deposition characteristics to soils and damage incurred by air pollutants on leaf cuticles, to field scale evaluations of industrial-generated dust on rare plants and habitats. The approach of much of this work is to apply biochemical and physiological tools to environmental problems.

**Michael J. Arbaugh** is a Forest Ecologist with the USDA Forest Service, Pacific Southwest Research Station in Riverside, California. He received a B.S. in Biology from the University of California, Riverside in 1980, an M.S. in Statistics from the University of California, Riverside in 1984, and a Ph.D. in Forest Ecology from Colorado State University, Ft. Collins in 1995. His research interests include understanding the multiple air pollutant effects on ecosystems, including large-scale patterns of air pollution and air pollution injury to western forests, long-term changes in forest composition, and passive air pollution monitor development and application in Class I areas.

**David Parker** is a Professor of Environmental Chemistry in the Department of Environmental Sciences at the University of California, Riverside. He earned a B.S. degree in Plant and Soil Science from the University of Vermont in 1978, an M.S. in Soil Science from Oregon State University in 1981, and a Ph.D. in soil chemistry from the Agronomy Department at Virginia Tech in 1988. He teaches courses in environmental sampling, biogeochemistry, and soil-plant interactions. His research interests include biogeochemistry and ecotoxicology of trace elements (Cd, Cu, Zn, Se) in soil-water systems, and the uptake and metabolism of potentially toxic elements in terrestrial plants.

**David Jones** is a Chemist with the USDA Forest Service, Pacific Southwest Research Station, Riverside, California. He has received his B.S. in Environmental Sciences from University of California in Riverside in 1990.
